# Supplementary material for: Proteome profiling in cerebrospinal fluid reveals novel biomarkers of Alzheimer's disease
Source: Mol Syst Biol. 2020 Jun 2;16(6):e9356. doi: 10.15252/msb.20199356 (PMC7266499; doi:10.15252/msb.20199356)
Supplement: Supplementary file 1 — Appendix [file MSB-16-e9356-s001.pdf]

# Appendix for

# Proteome Profiling in Cerebrospinal Fluid Reveals Novel Biomarkers of Alzheimer's Disease

Jakob M. Bader, Philipp E. Geyer, Johannes B. Müller, Maximilian T. Strauss, Manja Koch, Frank Leypoldt, Peter Koertvelyessy, Daniel Bittner, Carola G. Schipke, Enise I. Incesoy, Oliver Peters Nikolaus Deigendesch, Mikael Simons, Majken K. Jensen, Henrik Zetterberg, Matthias Mann

**Table of contents**

Appendix figure S1: page2

Appendix figure S2: page3

Appendix figure S3: page4

Appendix figure S4: page5

Appendix figure S5: page6

Appendix figure S6: page7

# Appendix Figures

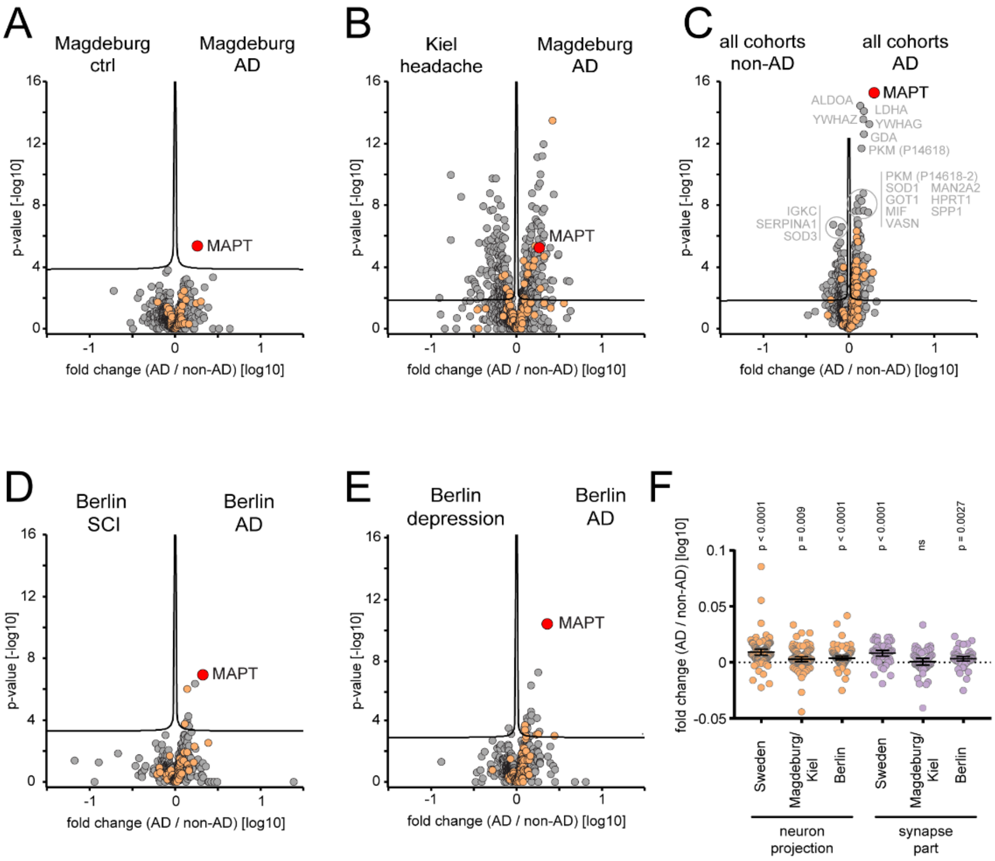


**Appendix Figure S1. Control subgroup-specific and global AD versus non-AD Volcano plots and neuronal signature analysis**

A-B) Volcano plots for AD versus non-AD comparisons within the Magdeburg/Kiel cohort, either using only the Magdeburg non-AD samples (A) or the Kiel non-AD samples as control group.

C) Volcano plots for a global comparison of AD versus non-AD CSF using all samples of the main study.

D-E) Volcano plots for AD versus non-AD comparisons within the Berlin cohort, either using only subjective cognitive impairment non-AD samples (D) or depression non-AD samples (E) as control group for AD.

F) Neuronal signature AD CSF association analysis for the three cohorts. AD versus non-AD fold changes are plotted for all proteins associated with the GO term neuron projection (orange, 95 proteins) and synapse part (violet, 55 proteins). Positive fold changes indicate enrichment in AD CSF over non-AD CSF, negative fold changes indicate enrichment in non-AD CSF over AD CSF. Bars represent mean and 95% confidence intervals of the mean. Statistical significance information for a difference from zero (equality in AD and non-AD CSF) calculated by a one sample t-test is given above the distributions, ns = not significant.

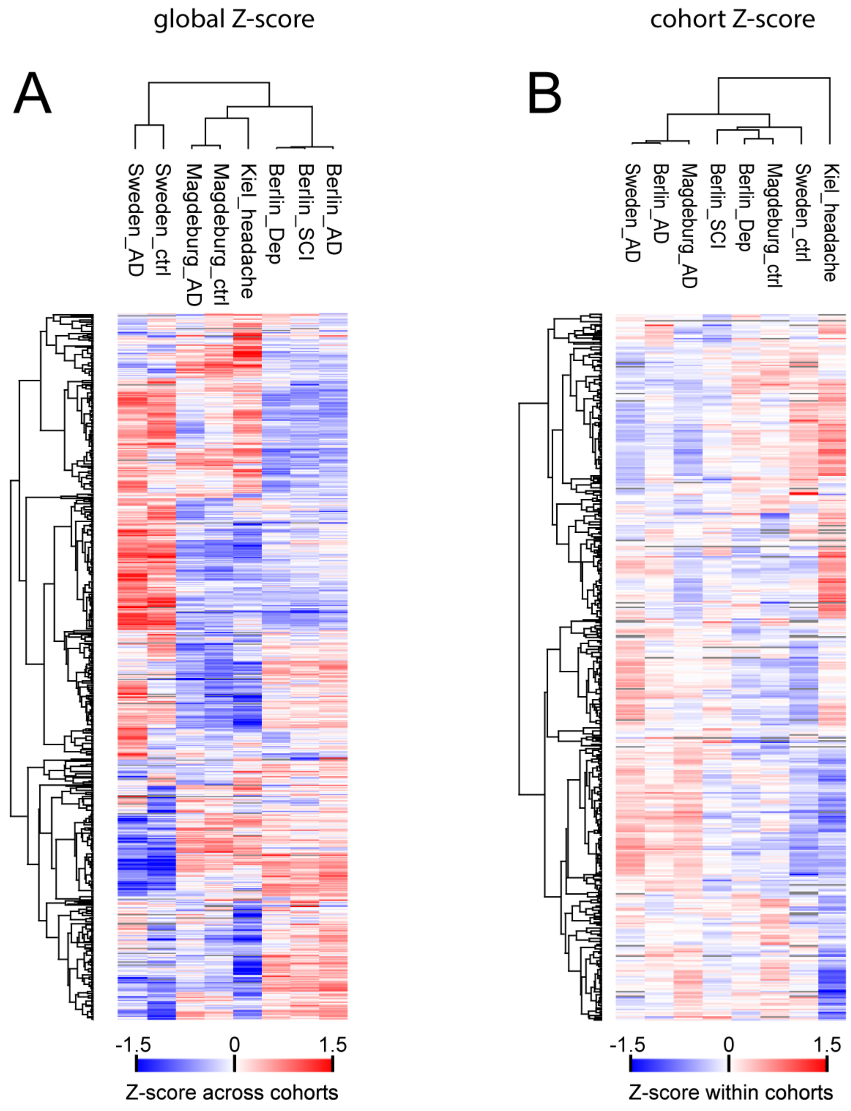


**Appendix Figure S2. Global CSF proteome clustering**

A) Clustering of the entire CSF proteome after global Z-scoring (across cohorts) separates cohort subgroups (AD and non-AD groups) by cohort.

B) Clustering of the entire CSF proteome after cohort Z-scoring (within cohorts each) separates cohort subgroups by AD versus non-AD status.

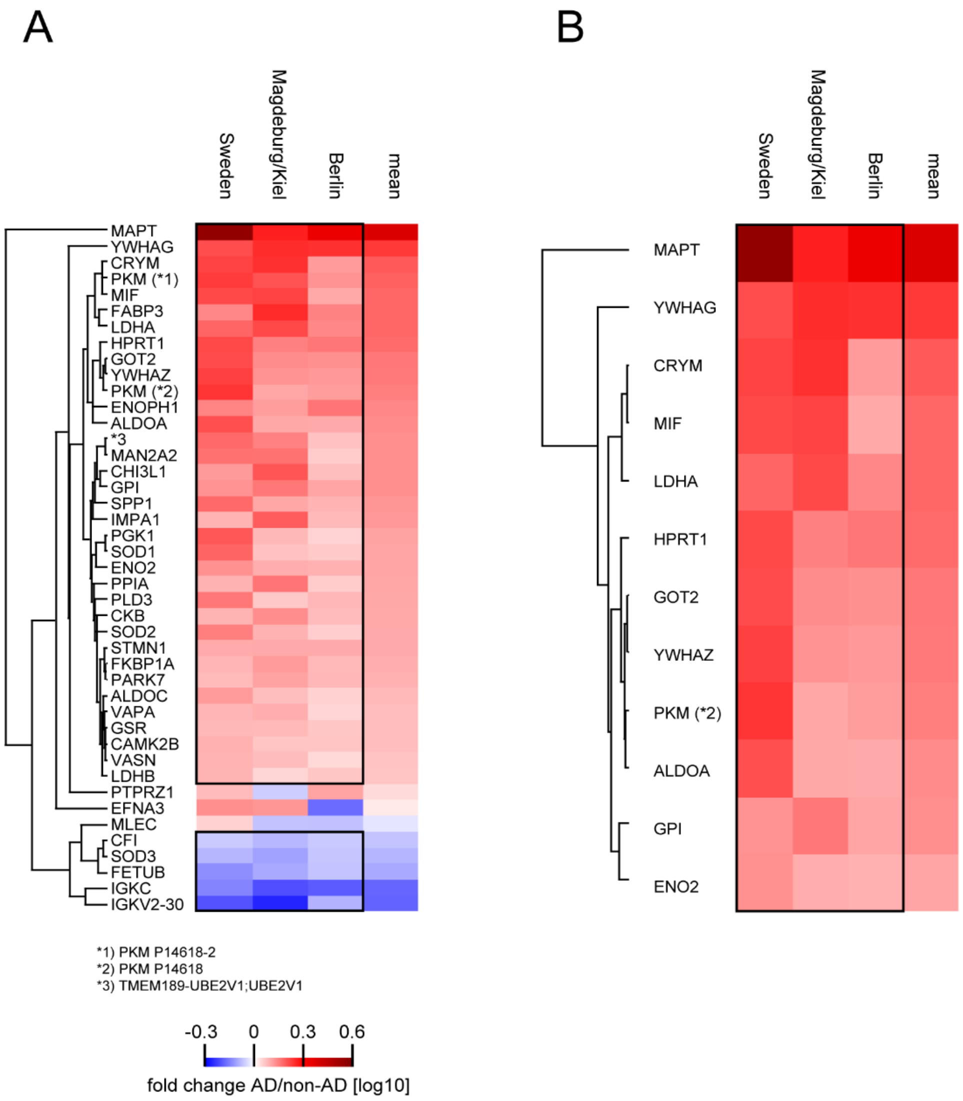


**Appendix Figure S3. Ranking of AD versus non-AD regulated proteins by fold change**

Heat map showing the AD/non-AD fold changes across three cohorts. Proteins included differ significantly ($p < 0.05$ in A, $q < 0.05$ in B) by AD status in each of the three cohorts. Proteins ranked by the mean fold change. Proteins with consistent fold changes highlighted by black boxes.

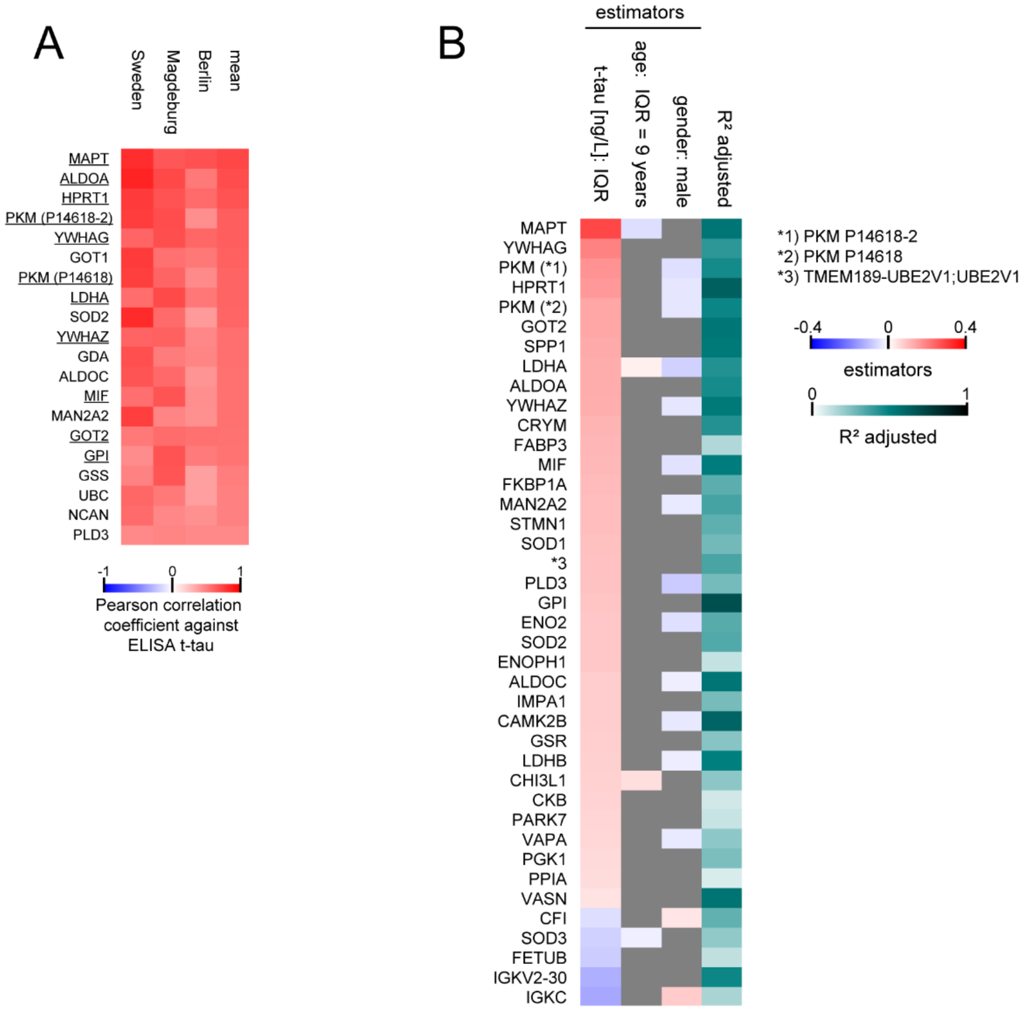


**Appendix Figure S4. Correlation and linear regression analysis against t-tau concentration**

A) Heat map showing the Pearson correlation coefficients of proteins correlating to t-tau significantly ($q < 0.05$) in each of the three cohorts. Underlined proteins also differ significantly ($q < 0.05$) in abundance by AD status in each of the three cohorts.

B) Linear regression model of protein intensity against t-tau concentration. Estimator strength for significant ($p < 0.05$) variables age and sex shown by heat map. In case of the continuous variables age and t-tau, the product of estimator and the inter-quartile range in this dataset shown as estimator strength. Overall explanation of variance by the regression model shown by $R^2$-values.

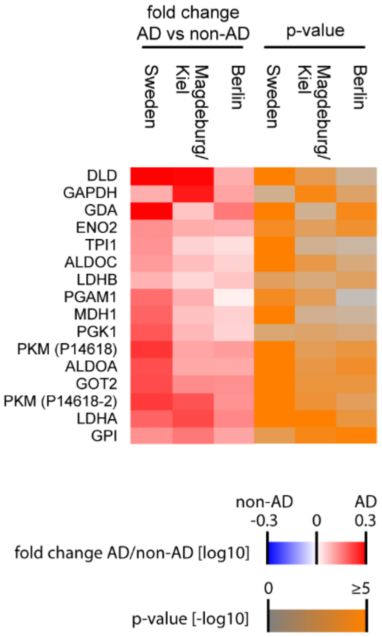


**Appendix Figure S5: AD-regulation of glycolytic proteins**
AD/non-AD fold changes shown for all quantified proteins with links to glycolysis or gluconeogenesis including those not significantly differing by AD status.

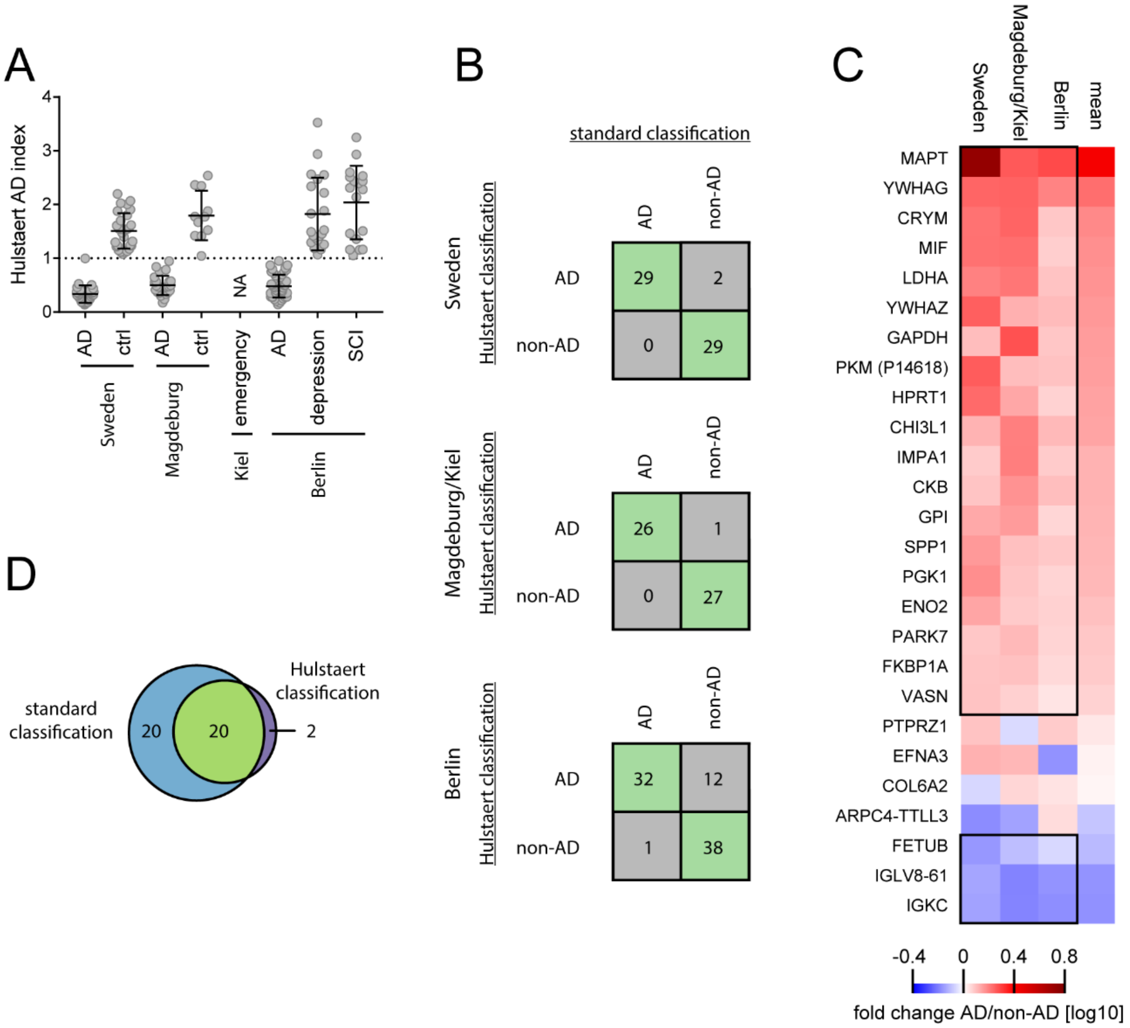


**Appendix Figure S6. Proteomics results when using the Hulstaert index for AD classification**

A) Hulstaert indices of AD and non-AD populations of this study.

B) Agreement between the Hulstaert index-based AD classification and our standard classification as described in the Materials and Methods section.

C) Intersection of significant ($p < 0.05$) proteins across the three cohorts of our study when using the Hulstaert index for AD classification. AD/non-AD fold changes in each cohort shown by the heatmap and proteins ranked according to the mean fold change.

D) Overlap of significantly ($p < 0.05$) and consistently AD-regulated proteins between the Hulstaert index and our standard AD classification
