# Supplementary material for: Proteome profiling in cerebrospinal fluid reveals novel biomarkers of Alzheimer's disease
Source: Mol Syst Biol. 2020 Jun 2;16(6):e9356. doi: 10.15252/msb.20199356 (PMC7266499; doi:10.15252/msb.20199356)
Supplement: Supplementary file 2 — Expanded View Figures PDF [file MSB-16-e9356-s002.pdf]

Expanded View Figures

Figure EV1. Clinical information on cohort subjects.

A Age of participants at the time point of CSF collection. Bars represent mean and standard deviation. Participant numbers are 29, 31, 26, 12, 16, 33, 26, and 24 for Sweden AD, Sweden biochemical controls, Magdeburg AD, Magdeburg biochemical controls, Berlin AD, Berlin depression controls, and Berlin subjective cognitive impairment (SCI) controls.

B–F CSF concentration of t-tau (B), p-tau_{181} (C), $A\beta_{1-42}$ (D), $A\beta_{1-40}$ (E), and the $A\beta_{1-42}/A\beta_{1-40}$ concentration ratio (F) as measured by ELISA. Bars represent mean and standard deviation. NA indicates that these data were not available. Participant numbers as in A) if data were available.

G–I CSF concentrations of t-tau plotted versus $A\beta_{1-42}$ for samples of the Sweden (G), Magdeburg (H), and Berlin (I) cohorts. Samples classified as AD according to the biochemical criteria of this study colored in red, samples as non-AD in blue.

J–K CSF concentration of t-tau plotted versus the $A\beta_{1-42}/A\beta_{1-40}$ concentration ratio for samples of the Magdeburg (J) and Berlin (K) cohorts. Samples classified as AD according to the biochemical criteria of this study colored in red, samples as non-AD in blue.

L Mini-mental state examination (MMSE) scores, a measure of cognitive performance. Bars represent mean and standard deviation. These data were only available for the Berlin cohort. Participant numbers are 33, 26, and 24 for Berlin AD, Berlin depression controls, and Berlin subjective cognitive impairment (SCI) controls.

 © 2020 The Authors

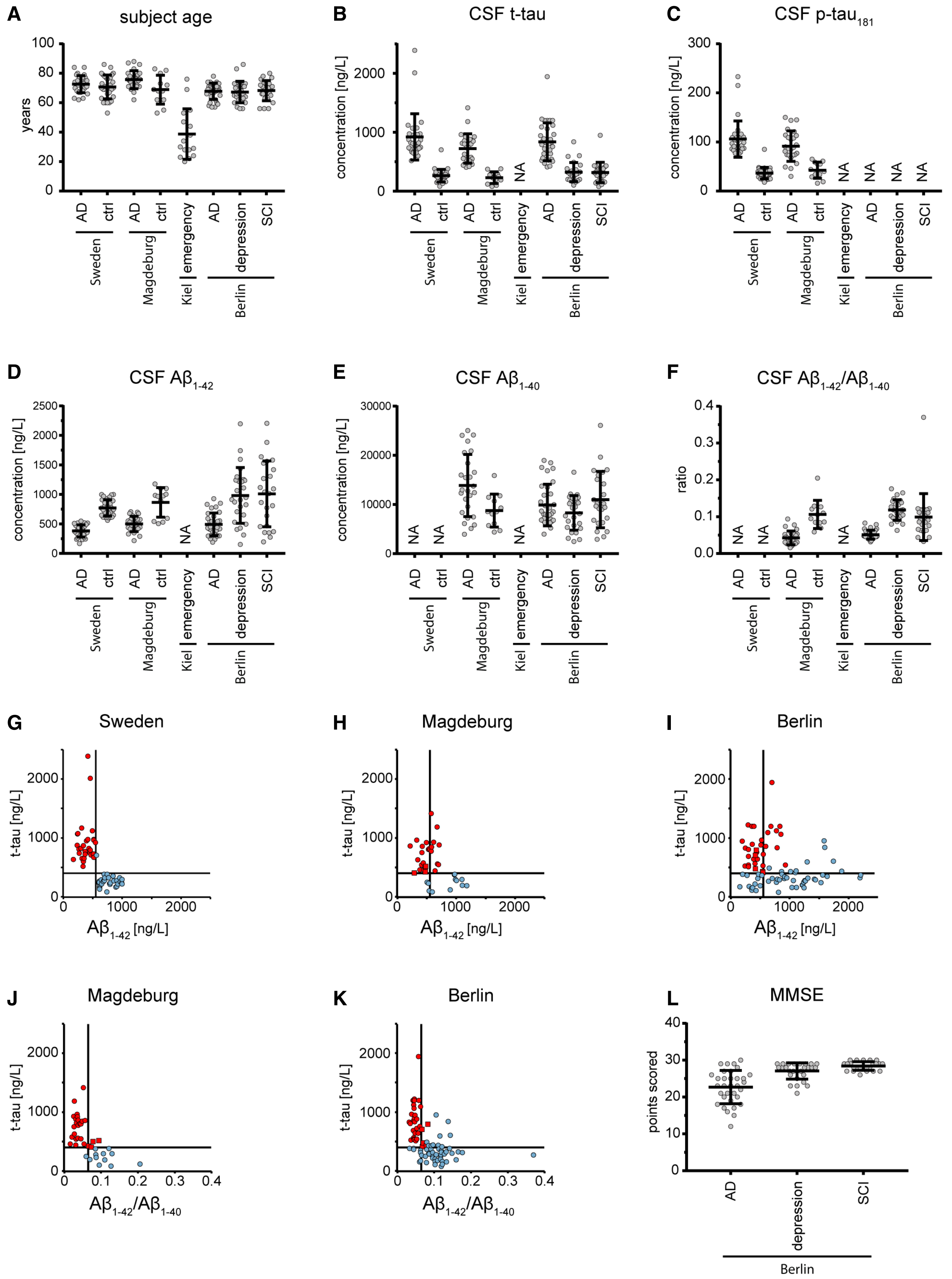
A subject age
B CSF t-tau
C CSF p-tau181
D CSF Aβ1-42
E CSF Aβ1-40
F CSF Aβ1-42/Aβ1-40
G Sweden
H Magdeburg
I Berlin
J Magdeburg
K Berlin
L MMSE
years
concentration [ng/L]
ratio
t-tau [ng/L]
Aβ1-42 [ng/L]
Aβ1-42/Aβ1-40
points scored
AD
ctrl
emergency
depression
SCI
NA
Sweden
Magdeburg
Kiel
Berlin

Figure EV1.

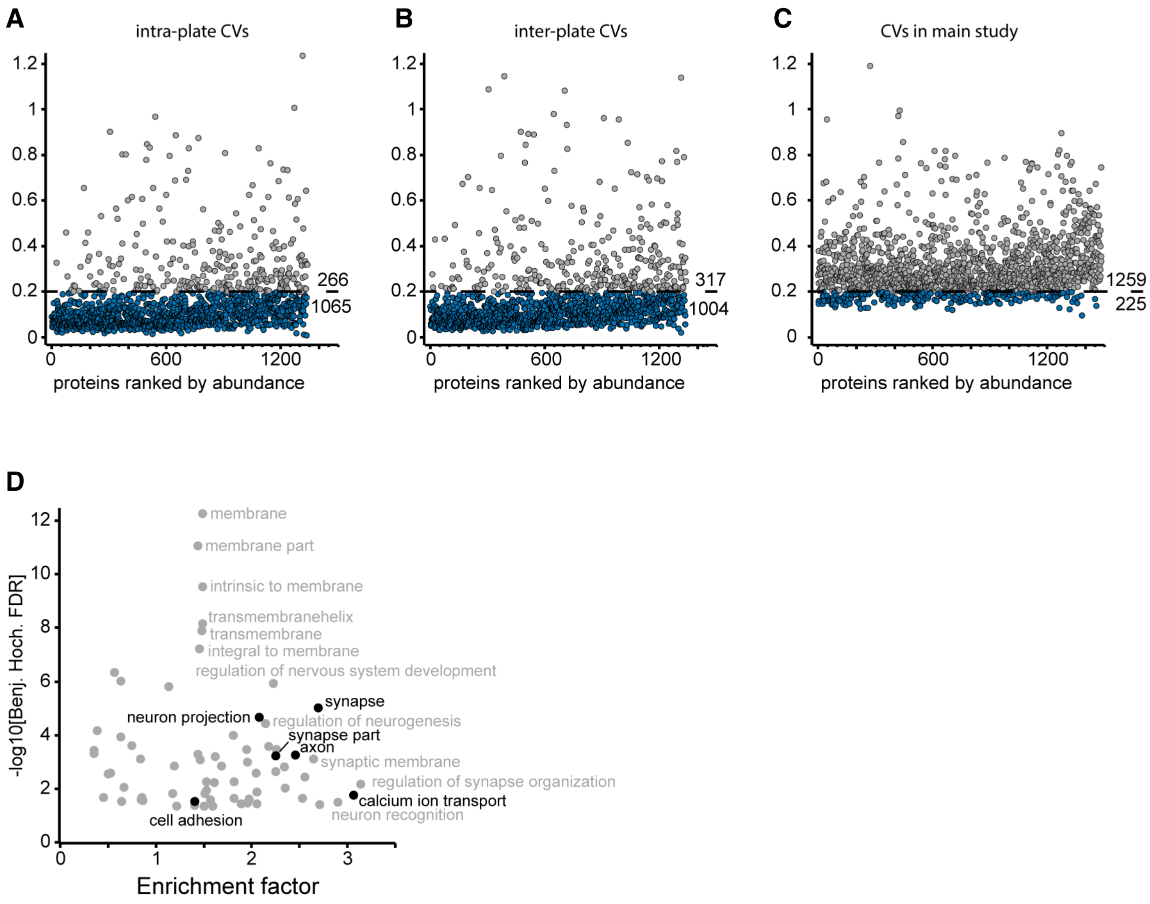

Figure EV2. Robustness of the analytical workflow and enrichment analysis of the tau-containing cluster in the global correlation map.

A–C Comparison of inter-participant variation and technical assay variation. Coefficients of variation (CVs) were determined in a separate experiment for intra-plate CVs (A) and inter-plate CVs (B) to benchmark protein quantitation. Biological CVs were calculated from the main study data (C). Proteins with a CV below 20% are highlighted in blue. Numbers of proteins above and below this CV cutoff are given above and below the cutoff line, respectively. The CV experiment data resulted from an independent protein search, and thus, the total number of identified proteins is not identical to the main study. The data show that technical variation is much smaller than inter-participant variation.

D Annotation enrichment results for the tau (MAPT)-associated cluster in Fig 1E. Enrichment in the cluster over the entire background CSF proteome vs. enrichment significance ($-\log_{10}$ of Benjamini–Hochberg-adjusted *P*-values). Terms of interest with links to neurons are highlighted in black.

Figure EV3. Effect of control group subtypes, age, and gender on the proteomics results.

A, B AD versus non-AD association of our 40-protein signature does not depend on the different control groups within the Berlin (A) and Magdeburg/Kiel (B) cohorts. AD versus non-AD fold changes for comparisons against the two distinct non-AD control groups, i.e. AD/depression vs. AD/subjective cognitive impairment (all Berlin) and AD/Magdeburg ctrl vs. AD/Kiel ctrl, are plotted for the Berlin and Magdeburg/Kiel cohorts.

C Linear regression model of protein intensity against AD/non-AD status. Estimator strength for significant ($P < 0.05$) covariates age and sex shown by heat map. In case of age, the product of estimator (per 1 year) and the interquartile range (eleven years) in this dataset shown as strength. Overall explanation of variance by the regression model shown by R^2 values.

▶

 © 2020 The Authors

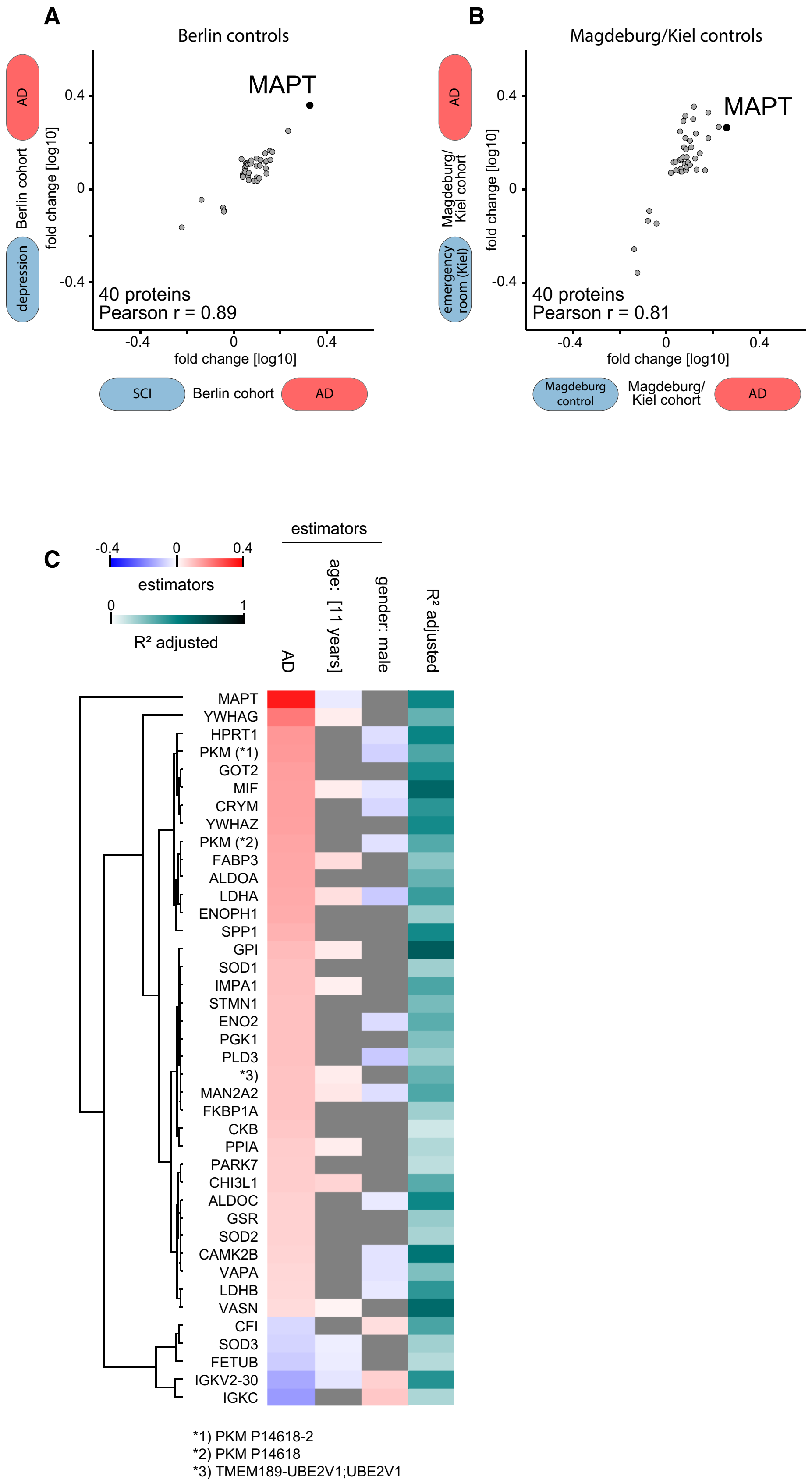
A
Berlin controls
MAPT
AD
depression
Berlin cohort
fold change [log10]
40 proteins
Pearson r = 0.89
SCI
Berlin cohort
AD
B
Magdeburg/Kiel controls
MAPT
AD
emergency room (Kiel)
Magdeburg/ Kiel cohort
fold change [log10]
40 proteins
Pearson r = 0.81
Magdeburg control
Magdeburg/ Kiel cohort
AD
C
estimators
R² adjusted
AD
age: [11 years]
gender: male
R² adjusted
MAPT
YWHAG
HPRT1
PKM (*1)
GOT2
MIF
CRYM
YWHAZ
PKM (*2)
FABP3
ALDOA
LDHA
ENOPH1
SPP1
GPI
SOD1
IMPA1
STMN1
ENO2
PGK1
PLD3
*3)
MAN2A2
FKBP1A
CKB
PPIA
PARK7
CHI3L1
ALDOC
GSR
SOD2
CAMK2B
VAPA
LDHB
VASN
CFI
SOD3
FETUB
IGKV2-30
IGKC
*1) PKM P14618-2
*2) PKM P14618
*3) TMEM189-UBE2V1;UBE2V1

Figure EV3.

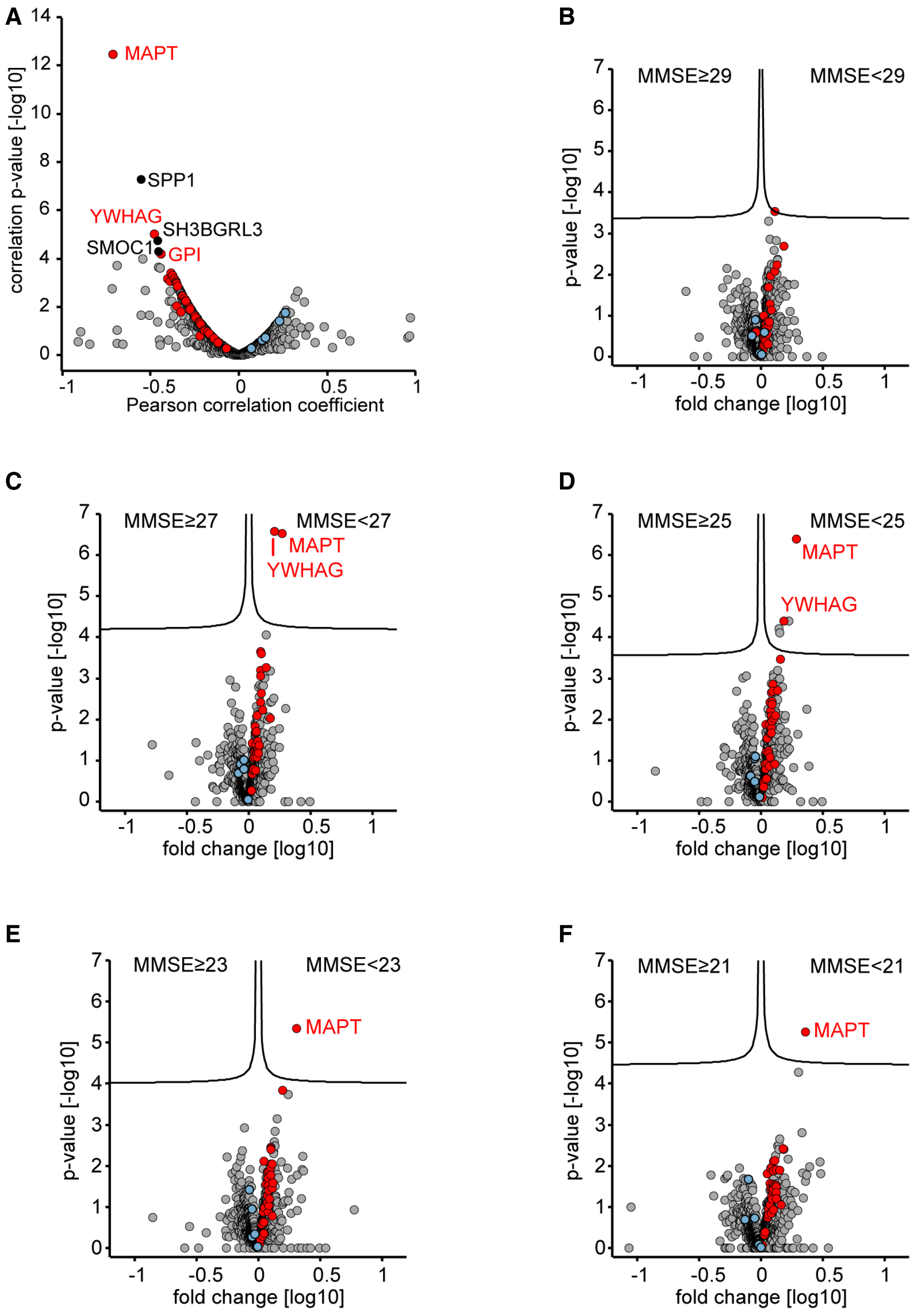

Figure EV4. MMSE score correlation analysis and proteome alterations when stratifying the Berlin cohort by MMSE score.

A Correlation of proteins to the mini-mental state examination (MMSE) scores in the Berlin cohort. Proteins with a correlation *q*-value below 0.05 are labeled. Proteins of the 40-protein signature are colored in red for proteins with increased abundance in AD CSF and in blue for proteins with increased abundance in non-AD CSF.

B–F CSF proteome alterations between groups of lower MMSE scores (poor neuropsychological performance) and groups of higher MMSE score as separated by cutoffs of 29 (A), 27 (B), 25 (C), 23 (D), and 21 (E). Proteins of the 40-protein signature are colored in red for proteins with increased abundance in AD CSF and in blue for proteins with increased abundance in non-AD CSF.

© 2020 The Authors

Figure EV5. Comparison of the results of our study with the CSF1 cohort of Higginbotham *et al*.

A Overlap of proteins identified as AD-regulated in the two studies. Out of the 43 proteins significantly ($P < 0.05$ across 3 cohorts) associated with AD or non-AD in this study, 40 exhibited consistent difference across 3 cohorts (40-protein signature). Of these, 38 were present in the CSF1 dataset of the Higginbotham *et al* study containing 2,875 proteins. In that dataset 528 proteins were significantly ($P < 0.05$) different between AD and control, 26 of the 38 matched proteins found as potential AD markers in this study overlapped. All 26 proteins had a consistent association with either AD or non-AD CSF across the CSF1 dataset and this study, 25 thereof were elevated in AD CSF and one protein elevated in non-AD CSF.

B Contingency table for analysis of enrichment of our three-cohort panel in the significantly AD vs. non-AD altered proportion of the CSF1 dataset.

C Heat map comparing the AD versus non-AD fold changes for the 26 protein intersection of significant ($P < 0.05$) proteins between our study and the CSF1 dataset. Proteins were ranked based on the mean fold change of our study.

D AD/non-AD fold changes of significant ($P < 0.05$) and consistent proteins of this study correlate well with fold changes of the CSF1 cohort.

© 2020 The Authors

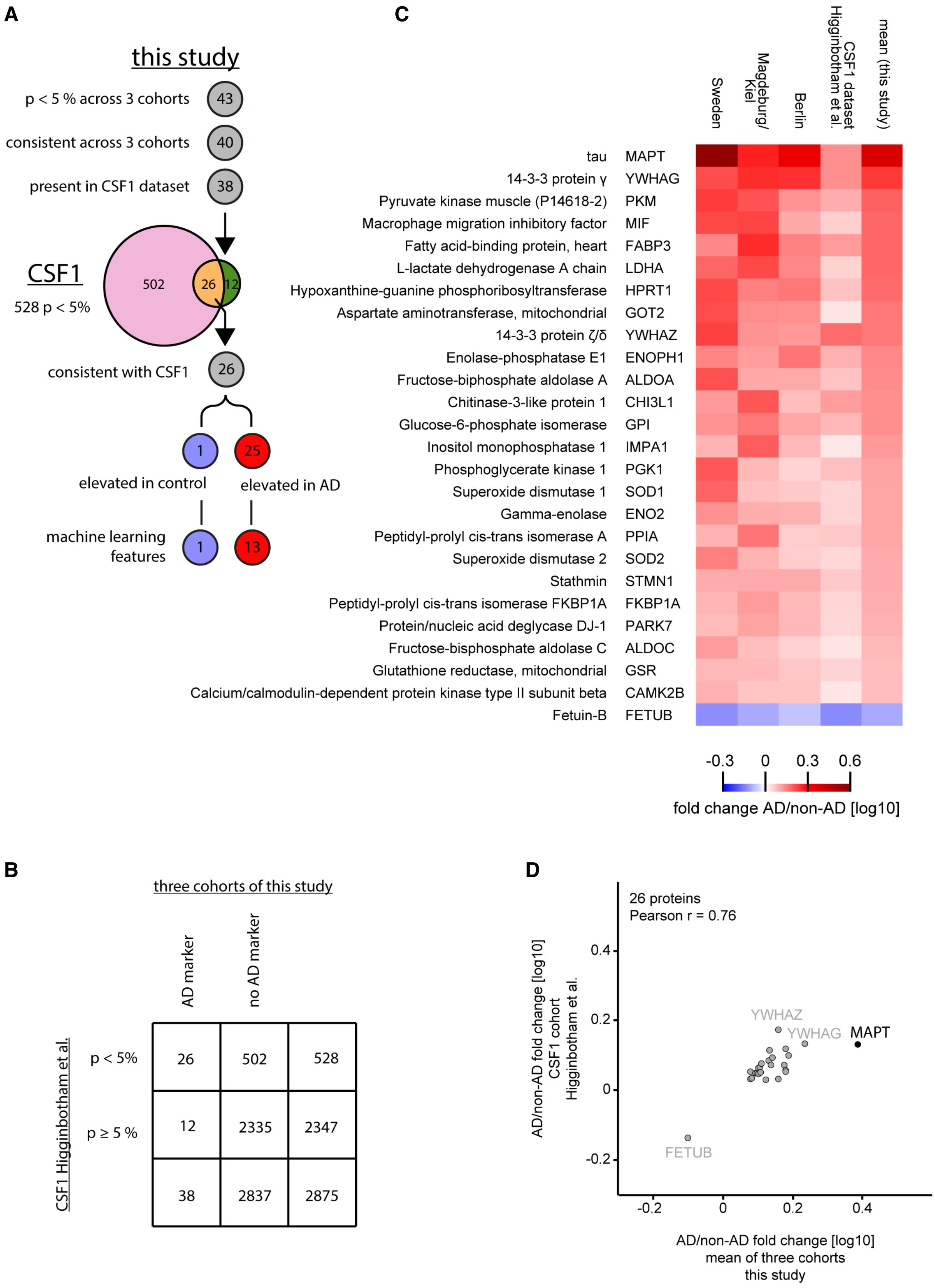

Figure EV5.

© 2020 The Authors
